# Evaluating self-reported vaccination hesitancy in mobile phone surveys in low- and middle-income countries: learned lessons from Ethiopia, Indonesia, Kenya, and Malawi

**DOI:** 10.7189/jogh.15.04066

**Published:** 2025-05-23

**Authors:** Ryan T Rego, Kyrani Reneau, Yuri Zhukov, Kristina Rice, Patrick Brady, Geoffrey Siwo, Ken Kollman, Sabina Odero, Mercy Mokaya, Amina Abubakar, Amy Pienta, Akbar K Waljee

**Affiliations:** 1 Center for Global Health Equity, Michigan Medicine, University of Michigan, Ann Arbor, Michigan, USA; 2 Clinton Health Access Initiative, Boston, Massachusetts, USA; 3 Inter-university Consortium for Political and Social Research, University of Michigan, Ann Arbor, Michigan USA; 4 School of Foreign Service and Department of Government, Georgetown University, Washington, D.C., USA; 5 Department of Learning Health Sciences, Michigan Medicine, University of Michigan, Ann Arbor, Michigan, USA; 6 Institute of Human Development, Aga Khan University, Nairobi, Kenya

## Abstract

**Background:**

The large amount of data on COVID-19 vaccination hesitancy presents a unique opportunity to better understand COVID-19 vaccination uptake. However, the utility of this data is unclear, particularly how representative the surveys are of general populations, how easy the data is to use, and how valid the outcome (intent to be vaccinated) is. We explored this in the World Bank’s high frequency phone surveys (HFPS).

**Methods:**

The HFPS were conducted longitudinally in over 50 countries between 2020–21. A subset of the HFPS contained questions on vaccination hesitancy. We compared the demographic results from four surveys against the most recent census to determine the representativeness of the sample and vaccination intent/actual vaccination against government-reported vaccination rates.

**Results:**

While the surveys were generally representative of population sizes and the rural/urban split, they tended to over-sample men and older people and omitted several key indicators. We also found that self-reported vaccination rates were higher than actual vaccination rates.

**Conclusions:**

It is important to consider challenges in the HFPS data and other datasets which measure vaccination acceptance by phone surveys. It is also important to consider the ease of data use. However, even when these challenges arise, there are still opportunities for meaningful use of the data.

The large amount of data on COVID-19 vaccination hesitancy presents a unique opportunity to better understand COVID-19 vaccination uptake. However, the utility of this data are unclear, particularly how representative the surveys are of general populations, how easy the data are to use, and how valid the outcome (intent to be vaccinated) is. We explored this in the World Bank’s high-frequency phone surveys (HFPS). The HFPS were conducted longitudinally in over 50 countries between 2020–21. A subset of the HFPS contained questions on vaccination hesitancy. We compared the demographic results from four surveys against the most recent census to determine the representativeness of the sample and vaccination intent/actual vaccination against government-reported vaccination rates. While the surveys were generally representative of population sizes and the rural/urban split, they tended to over-sample men and older people and omitted several key indicators. We also found that self-reported vaccination rates were higher than actual vaccination rates. It is important to consider challenges in the HFPS data and other data sets which measure vaccination acceptance by phone surveys. It is also important to consider the ease of data use. However, even when these challenges arise, there are still opportunities for meaningful use of the data.

Despite more than two-thirds of the world’s population receiving at least one dose of the COVID-19 vaccine, less than a quarter of people in low-income countries have received at least one dose of the vaccine [1]. This disparity in vaccination poses a tremendous challenge to preventing morbidity and mortality from COVID-19, avoiding the proliferation of new variants, and ultimately ending the pandemic [2]. As global vaccine supply chains continue to strengthen and provide increasing supplies of vaccinations to low- and middle-income countries (LMICs), vaccination hesitancy is becoming the main driver of vaccination disparities [3]. We define vaccination hesitancy as refusal or delay in vaccination. While hesitancy is a global issue, the availability of tools and knowledge to combat hesitancy are not available in a globally equitable manner. For example, Pires conducted a review of all papers measuring hesitancy to the COVID-19 vaccination globally – finding only seven papers from LMICs (and 30 from high-income countries) [4]. Since Pires’ search, a handful of additional studies have emerged in LMICs in recent months, but the evidence base still remains lacking – particularly in the lowest-income countries and countries without good research infrastructure. As such, significant work is still urgently needed to understand the extent and causes of vaccination hesitancy and how this may differ across countries and contexts, especially considering the continuing low vaccination rates among many LMICs.

Utilising pre-existing data on vaccination hesitancy and self-reported vaccination offers a tremendous opportunity to create knowledge to combat vaccination hesitancy. Various actors have collected large amounts of data in LMICs since the beginning of the pandemic. One actor is the World Bank Group, which ran mobile phone surveys in 53 countries to measure both hesitancy to COVID-19 vaccination and self-reported COVID-19 vaccination [5]. Although the availability of these data creates an opportunity to better understand hesitancy and improve vaccination rates in LMICs, it is also important to acknowledge the data’s limitations.

There are several limitations to the World Bank Groups data to note, which we discuss at length later in this paper, including: 1) the data are self-reported and subject to biases, including social-desirability bias, recall bias, and the Hawthorne effect, 2) the data may not adequately represent the entire population, especially considering that it is collected by mobile phone survey, and 3) World Bank Group data collection was not performed with the intention for use in vaccination hesitancy studies and therefore, the underlying survey study designs were potentially not optimal for vaccination hesitancy studies. A recent study using rounds of high frequency phone surveys (HFPS) data from Burkina Faso, Ethiopia, Malawi, Nigeria, and Uganda found that women, better-educated individuals, and those living in better-off households are more likely to express vaccine hesitancy [6], noting how purposive selection can lead to over-representation of certain populations. In examining HFPS from Ethiopia, Malawi, Nigeria, and Uganda Ambel et al. found that overall bias was skewed towards urban and wealthier households. Using the same HFPS, Brubaker et al. concluded that respondents are more likely to be household heads and males compared to the general population [7,8]. The authors suggest applying survey weight adjustments using information from face-to-face surveys to reduce bias [7] yet this technique does not eradicate bias on all levels [8]. As with other data sources, it is also important to consider possible biases introduced by analyses, particularly conformational bias.

In this paper, we examined four World Bank Group data sets on vaccination hesitancy from Malawi, Kenya, Ethiopia, and Indonesia to explore potential biases in self-reported vaccination hesitancy and vaccination data obtained from surveys and their analysis and to explore methods through which these biases can be accounted for and understood. These countries were chosen given the ability to analyse data at the level two and three geographic levels, wide coverage throughout the countries, and a large sample size of the data sets compared to other HFPS data sets. With this, we hope that others will be able to use these data in a meaningful and rigorous manner and that those interpreting their results can do so while taking into account the context of possible biases.

## METHODS

### Description of the data sources

We evaluated publicly available, de-identified data from the World Bank Group’s HFPS in Malawi, Kenya, Ethiopia, and Indonesia (**Table 1**). The World Bank began collecting data for these longitudinal surveys in 2020 at the beginning of the COVID-19 pandemic in over eighty contexts. While the main objective of these studies was to measure the economic impact of COVID-19, 53 of the surveys included questions on both intent to receive a COVID-19 vaccination and actual COVID-19 vaccination. Of these surveys, we chose to analyse the surveys from Indonesia, Kenya, and Malawi, as they were larger surveys with rigorous sampling methods that attempted to represent the general population and were from three distinct regions (East Africa, Southern Africa, and South-East Asia). They also contained granular geographic information, which may be useful for other analyses. These surveys all contained questions on vaccination hesitancy, including self-reported vaccination intent ‘if the vaccine was available to you now at no cost, would you take it’ and select questions on beliefs towards vaccinations and motivators and barriers towards vaccination. For all surveys, replacements were sought for dropouts between rounds. More information on the data source, including measurement methodologies and ethics, is available from World Bank Microdata Libraries, referenced below. We did not seek ethics approval as this was an analysis of secondary, de-identified data.

**Table 1:** Summary of surveys included in our analysis

Country	Time frame	Sample, n	Outcome
Malawi	May 2020–May 2022 (12 rounds)	2337	Vaccination intent (rounds 5 and 8)
Kenya	May 2020–July 2022 (6 rounds)	9000	Vaccination intent (rounds 3–6)
Ethiopia	April 2020–June 2021 (12 rounds)	5374	Vaccination intent (rounds 6 and 10)
Indonesia	May 2020–April 2022 (7 rounds)	4338	Vaccination intent (rounds 4 and 5), self-reported vaccination (rounds 6 and 7)

### Malawi HFPS

The Malawi HFPS were collected longitudinally from a sample of 2337 households over 12 rounds between May 2020 and May 2022, with 1400–1800 responding each round. Rounds five and eight contained questions on vaccination intent (with no rounds questioning actual vaccination). The survey also asked why those who did not want to be vaccinated did not [9].

### Kenya HFPS

The Kenyan HFPS were collected longitudinally from a sample of 9000 households over six rounds between May 2020 and July 2022, with 4000–5000 responding each round. The last four rounds contained questions on vaccination intent (with no rounds questioning actual vaccination). The survey also asked in round four why those who did not want to be vaccinated did not want to be vaccinated and about willingness to pay for vaccination in rounds four and six [10].

### Ethiopia HFPS

The Ethiopian HFPS were collected longitudinally from a sample of 5374 households over 12 rounds between April 2020 and June 2021, with 800–3200 responding each round (decreasing as time progressed). Rounds six and 10 contained questions on vaccination intent (with no rounds questioning on actual vaccination). The survey also asked why those who did not want to be vaccinated did not [11].

### Indonesia HFPS

The Indonesian HFPS were collected longitudinally from a sample of 4338 households over seven rounds between May 2020 and April 2022, with 3000–4000 responding per round. The last four rounds asked questions on vaccination – rounds four and five on intent using the aforementioned question, rounds six and seven on self-reported vaccination, asking ‘have you received the COVID-19 vaccine’ (subsequently questioning on the number of doses received, if any). In rounds four and five, if the respondent was not willing to be vaccinated, they were asked why. In rounds six and seven, if they were not vaccinated, they were asked if they were willing to be vaccinated, and if not, why so [12].

### Variables and analysis

We used variables on the following demographics: the number of households (based on the weighted number of households), age of the respondent, gender of the respondent, education of the respondent, urban/rural status, and household size. For vaccination, in Kenya, Ethiopia, and Malawi, we analysed responses to vaccination intent (question-wording: ‘If the vaccination was available for you at no cost, would you take the vaccination’) in the most recent survey round available. In Indonesia, we examined actual vaccination (question wording: ‘Have you received at least one dose of the COVID-19 vaccination’) in the most recent survey round available. We conducted univariate analyses of these from the HFPS by tabulating the outcomes while applying population-level weights. We then qualitatively compared these results to census data and government reported vaccination data. Weights provided by the World Bank were used at the household level, and can be found on the respective HFPS sources.

### Description of the data sources

#### Census data

To determine how representative the HFPS’ were of the true population, we pulled data on key demographics from the most recent census in the four countries. In Malawi, the most recent census was conducted in 2018 by the National Statistics Office of Malawi. In Kenya, the most recent census was conducted in 2019 by the Kenya National Bureau of Statistics. In Ethiopia, the most recent census was conducted in 2007 by the Ethiopian Statistics Service, and in Indonesia, the most recent census was conducted in 2020 by Statistics Indonesia [13–16].

#### Vaccination data

To determine how close the HFPS’ vaccination data was to actual vaccination, we pulled vaccination data from Our World in Data [17]. Our World in Data updates its data sets daily with vaccination data from national-level ministries of health. We pulled actual vaccination numbers on 21 August 2022, well into the period when vaccinations were available to the entire population in all four countries. COVID-19 vaccination rollout was as follows: 13 January 2021 (Indonesia), 4 March 2021 (Kenya), 11 March 2021 (Malawi), and 13 March 2021 (Ethiopia) [18–20].

## RESULTS

### Representativeness of the true population

Through a weighted, univariate analysis of the HFPS data we estimated numbers and rates of key demographic indicators. We also extracted the same key demographic indicators from most recent census data from each country (Table S1 in the **Online Supplementary Document**).

### Kenya

In Kenya, the number of households in the weighted HFPS sample was close to that in the 2019 census, just a 1% difference. However, the HFPS under sampled females, older segments of the population, those in urban settings, and smaller households. We were unable to find data from the census on education in Kenya, so cannot speak to the performance of the HFPS in being representative of education levels in Kenya.

### Indonesia

In Indonesia, the number of households in the weighted HFPS was close to that of the 2020 census, a 2% difference. However, the HFPS drastically under sampled women compared to the census and also skewed older. The urban/rural split in the HFPS was close to that of the census. The census did not include data on education, and HFPS did not include data on household size, so we were unable to compare those demographics.

### Malawi

In Malawi, the number of households in the weighted HFPS was 10% lower than that of the 2018 census. The HFPS under sampled females and skewed older. The urban/rural split was similar between the HFPS and census, though the HFPS slightly oversampled larger households. There was no data on education in either the HFPS or the census.

### Ethiopia

In Ethiopia, the HFPS contained nearly 30% more households than the 2007 census, though this is understandable given the time that has elapsed. The census also under sampled females and skewed older. As the census did not include information on urban/rural split, education, or household size, we could not make those comparisons (though given the age of the census, they likely would not be representative of 2020 Ethiopia regardless).

### Overall trends

Overall, we saw that while the HFPS was generally representative of the number of households in each country, it oversampled males and older people. We discuss possible reasons for this and its implications in the discussion.

### Self-reported vaccination or vaccination intent compared to actual vaccination

Through a weighted, univariate analysis of the HFPS data, we estimated self-reported vaccination intent (for Kenya, Malawi, and Ethiopia) and self-reported vaccination of at least one dose and compared it to data from Our World in Data pulled at the end of August 2022 (Table S2 in the **Online Supplementary Document**). We found that for all countries, self-reported vaccination intent and vaccination were higher than actual vaccination rates well into the period when the entire population had vaccinations available.

## DISCUSSION

By comparing four of the World Bank Group’s HFPS to census and government-issued vaccination data, we have demonstrated that issues need to be accounted for when using secondary, self-report data collected by mobile phone. While the surveys we analysed were representative of population sizes and rural/urban split, they tended to over-sample men and older people. Further, several key indicators were missing (though we will discuss methods to mitigate this). We also found that self-reported vaccination data (both on intent and actual vaccination) was far higher than actual vaccination rates far after the collection of the self-report data when there was ample opportunity to be vaccinated. We will also discuss reasons for this and methods to mitigate.

### Issues in population representativeness

We found that the HFPS generally over sampled males and older people. Considering that the HFPS was a mobile phone survey, this may be due to differences in mobile phone ownership. Though we are unable to find studies on mobile phone ownership among those aged <18 years in LMICs, we assume that ownership is higher in adults than adolescents and perhaps in urban areas than rural areas (though we generally saw good representation of the rural population). Indeed, if you are to calculate the mean age excluding anybody aged <18 years, you then get ages much closer to that of the HFPS. For example, the mean age of the HFPS sample was 38, with the mean age of Kenyans >18 years being 38 (per the census), and there were similar trends in the other four countries. However, this does not account for the issues we found related to gender representation. The Kenyan HFPS contained 10 percentage points (pp) more males than females, despite there only being a four pp difference in mobile phone ownership [21]. Similarly, in Malawi, there is a 14 pp gap in ownership between males and females but a 25 pp gap in the HFPS. In Indonesia, there is only an eight pp gap in ownership but a 35 pp gap in the HFPS [22]. While it is worth noting that in Indonesia the gender gap in the HFPS was less than that of the mobile phone ownership gap (25 pp *vs.* 36 pp), this does not discount the need to better include women [23]. However, considering that there was no ground truth of the results, we cannot be sure that mobile phone ownership was the only cause of issues in population representativeness. Other alternative hypotheses include issues in the survey’s sampling strategy, such as time of calling or how sampling frames were drawn, as well as biases in who consents to take part in the survey after being contacted due to the time burden. It is also worth noting that the HFPS did not include other important demographic indicators, such as sexual orientation, disability, and migratory status.

Under sampling of certain groups and the omission of indicators on other groups creates key considerations for data use. When under sampling, one runs the risk of drawing conclusions that are not actually generalisable to the entire population, but rather the oversampled group. However, when not including variables on marginalized populations, it is not possible to use a single data set to examine behaviours in these populations. This is critical as marginalised populations often have differing behaviours and needs from the general population. However, we may be able to partially remedy this through augmentation with other data sets, a method which we discuss in our accompanying paper.

### Misalignment between vaccination intent/report in HFPS and actual data

We also estimated that reported rates of vaccination intent (and for Indonesia, self-reported actual vaccination) in the HFPS were far higher than actual government-reported vaccination rates, even well after the HFPS surveys were conducted to ensure that all respondents had ample opportunity to be vaccinated. We hypothesise two main reasons for this – social desirability bias and access. When surveying health topics, social desirability bias is often of concern when there is a response which is clearly socially desirable [24–26]. While one may expect assurance of confidentiality (compounded by the inherent privacy of phone interviews) to counteract this, we have demonstrated that this may not be the case [27]. Indeed, Wolter et al. found in Germany that direct questioning on COVID-19 vaccination led to a 10 pp higher reported vaccination rate than indirect questioning using the item count technique [28]. We suspect this to also be the case for vaccination intent, particularly when interacting with cultural norms (which require further exploration. It is also important to consider that attitudes towards vaccines may continue to change over time, another potential explanation for the discrepancies between intent to vaccinate and actual vaccination rates [6]. Indeed, Fridman et al. found that in the USA COVID-19 vaccination intention went down when the vaccine became available – with another study from the UK finding evidence that this is due to concerns around potential risks of vaccination increasing over time [29,30]. Further, access to vaccination may also be an issue, which explains our results. Access to vaccination has been shown to be a significant factor in gaps between vaccination intent and actual vaccination [31,32]. However, considering that vaccines are readily available in all four countries, we expect this bias to be minimal. We explore the gap between vaccination intent and actual vaccination (measured through self-report) in our forthcoming paper based on the Indonesian HFPS.

The Indonesia HFPS questioned on actual vaccination. While this indicator is closer to the government-reported vaccination rate, it is important to consider biases in this indicator. In addition to social desirability bias, recall bias and the Hawthorne effect may be at play. Considering the relative recency of vaccination programs, we assume that the effects of recall bias are minimal, but as the participants were questioned multiple times since the start of the pandemic, they may have adapted their health behaviours or beliefs as a result of the questioning, leading to increased vaccination. Indeed, Polgreen et al. found evidence of the Hawthorne effect for the influenza vaccine among physicians in the USA, where being observed was associated with an increase in vaccination without direct intervention [26,33]. As such, while the vaccination numbers reported in the HFPS may be accurate to the respondents, they may not be representative of the general population. However, we may be able to partially remedy the issues of inaccurate vaccination numbers by combining the HFPS data with government-collected actual vaccination data, which we discuss in the accompanying paper.

### Other considerations

When analysing these data, there are several other considerations which became apparent to us. The first is to construct rigorous, theoretically supported analysis plans which do not fall into the traps of conformational bias or data manipulation. Given the relatively limited amount of data on vaccination hesitancy in LMICs, this may include thorough exploratory analyses. Second, when using secondary data, it is important to ensure that thorough support is available to assist with the recording, weighting, and interpretation of the data. Thankfully, the World Bank team has support available for many of its data sets. Finally, it is worth considering the general ease of use of the data - is it available in a combined, recoded format, or are these and other transformations required? We explore some transformations, primarily the combination of these data with other data on political and economic factors at the geospatial level in another paper [34].

### Limitations

It is important to recognise some limitations in our analysis, mostly stemming from the reliability of the census and government vaccination data. First, the census data often is not from the same time period as the administration of the HFPS, particularly in the case of Ethiopia, where there is a 15-year gap. Demographics may have changed substantially in this period. Second, census data may still not be accurate, given the underrepresentation of certain groups in conflict-affected regions, groups who are unregistered, and other factors. Third, given the limited amount of information in both census and HFPS data, it is impossible to compare several important demographic indicators, as described above. Fourthly, the data we report are exclusive to the household head and likely skewed towards males and older people. The survey was also conducted by mobile phone, likely skewing towards more educated and higher income groups [7,8]. Finally, it is also important to consider the reliability of the government-provided vaccination data. While one may reasonably assume that government vaccination data are reliable, considering that it is the latest reported number of vaccinations nationally, underreporting does occur. Vaccinations may not be reported due to failures in health information systems, vaccinations through unofficial channels, and simply unrecorded vaccinations. Further, it is possible (on a much lower scale) that vaccinations are overcounted due to misrecording of second doses as a first dose or corruption, problems hypothesised in all countries (not just LMICs) [35,36]. We are also unable to critically consider all structural barriers to vaccination.

## CONCLUSIONS

We have demonstrated that while mobile phone collected secondary data presents tremendous opportunities to better understand vaccination hesitancy, key considerations must be made. Data may oversample males and older populations; and there may be biases in reported rates of both vaccination and vaccination hesitancy. We recommend that future research focus on collecting data through ways that are not as prone to population misrepresentation and bias in outcomes or examine ways of combining data with other more reliable data sources, which we are currently exploring in the accompanying paper.

## Additional material


Online Supplementary Document
