# Supplementary material for: Evaluating self-reported vaccination hesitancy in mobile phone surveys in low- and middle-income countries: learned lessons from Ethiopia, Indonesia, Kenya, and Malawi
Source: J Glob Health. 2025 May 23;15:04066. doi: 10.7189/jogh.15.04066 (PMC12100674; doi:10.7189/jogh.15.04066)
Supplement: Online Supplementary Document [file jogh-15-04066-s001.pdf]

Supplement to: Rego RT, Reneau K, Zhukova Y, Rice K, Brady P, Siwo G, Kollman K, Odero S, Mokaya M, Abubakar A, Pienta A, Waljee AK. Evaluating self-reported vaccination hesitancy in mobile phone surveys in low- and middle-income countries: learned lessons from Ethiopia, Indonesia, Kenya, and Malawi. J Glob Health. 2025;15:04066.

Table S1. Key demographic indicators from HFPS and censuses

	Kenya		Indonesia		Malawi		Ethiopia	
**Indicators**	**May–July 2022**	**2019 census**	**April 2022**	**2020 census**	**February–March 2021**	**2018 census**	**February 2021**	**2007 census**
Households (n)	11 947 522	12 143 913	71 438 289	73 100 000	3 567 435	3 984 986	19 857 863	15 600 000
Females (%)	40.5	50.5	14.2	49.4	24.2	51.5	25.7	50.1
Mean age in years ($\bar{x}$)	37.9	19.1	47.8	29.7	45.0	18.9	38.7	17.9
Urban (%)	37.5	31.2	61.9	57.9	19.6	16.0	ND	16.0
Secondary or over education (%)	71.6	ND (for adult population)	34.65	ND (for adult population)	ND	ND (for adult population)	ND	ND (for adult population)
Household size ($\bar{x}$)	2.0	3.9	ND	3.9	5.4	4.4	ND	4.8

ND – no data, $\bar{x}$ – mean

Table S2. Vaccination intent and rates from HFPS and Our World in Data*

	Kenya		Indonesia		Malawi		Ethiopia	
Statistic type	**May–July 2022**	**Actual**	**April 2022**	**Actual**	**February–March 2021**	**Actual**	**February 2021)**	**Actual**
Actual vaccination†	NA	23.5	87.0	74.2	NA	12.9	NA	37.5

Self-reported intention vaccination	88.3	NA	52.4	96.6

NA – not available

*Presented as %.

†At least one dose; self-reported in HFPS.
